# Supplementary figures and images for: Plant dieback under exceptional drought driven by elevation, not by plant traits, in Big Bend National Park, Texas, USA
Source: PeerJ. 2014 Jul 15;2:e477. doi: 10.7717/peerj.477 (PMC4106195; doi:10.7717/peerj.477)

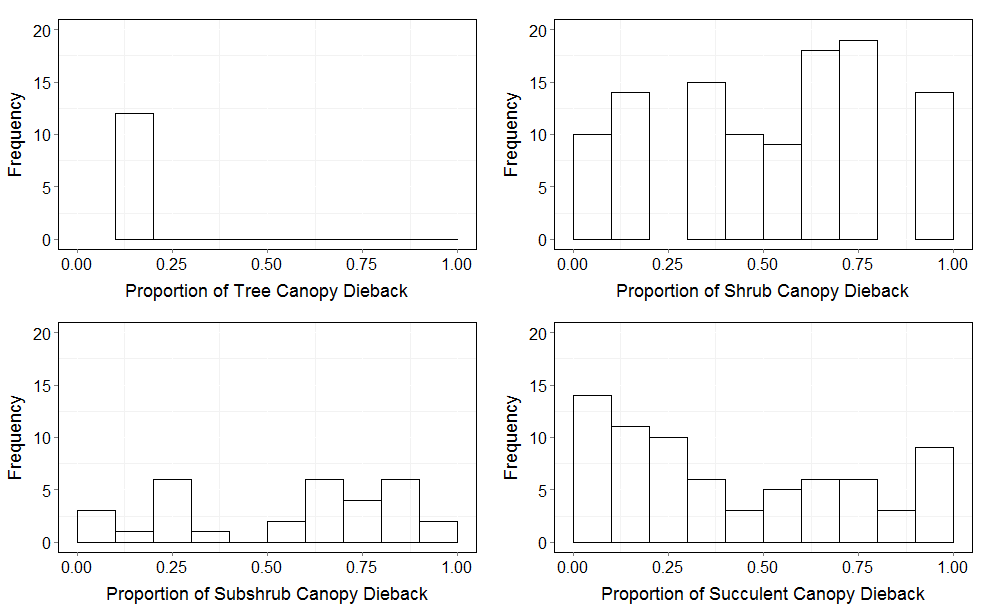

Supplement: Supplemental Information 1 — Frequency distribution of dieback for all elevations for each growth form from 2011. The growth forms are shown starting in the upper left corner and moving clockwise as: tree, shrub, succulent, and subshrub. [file peerj-02-477-s001.png]

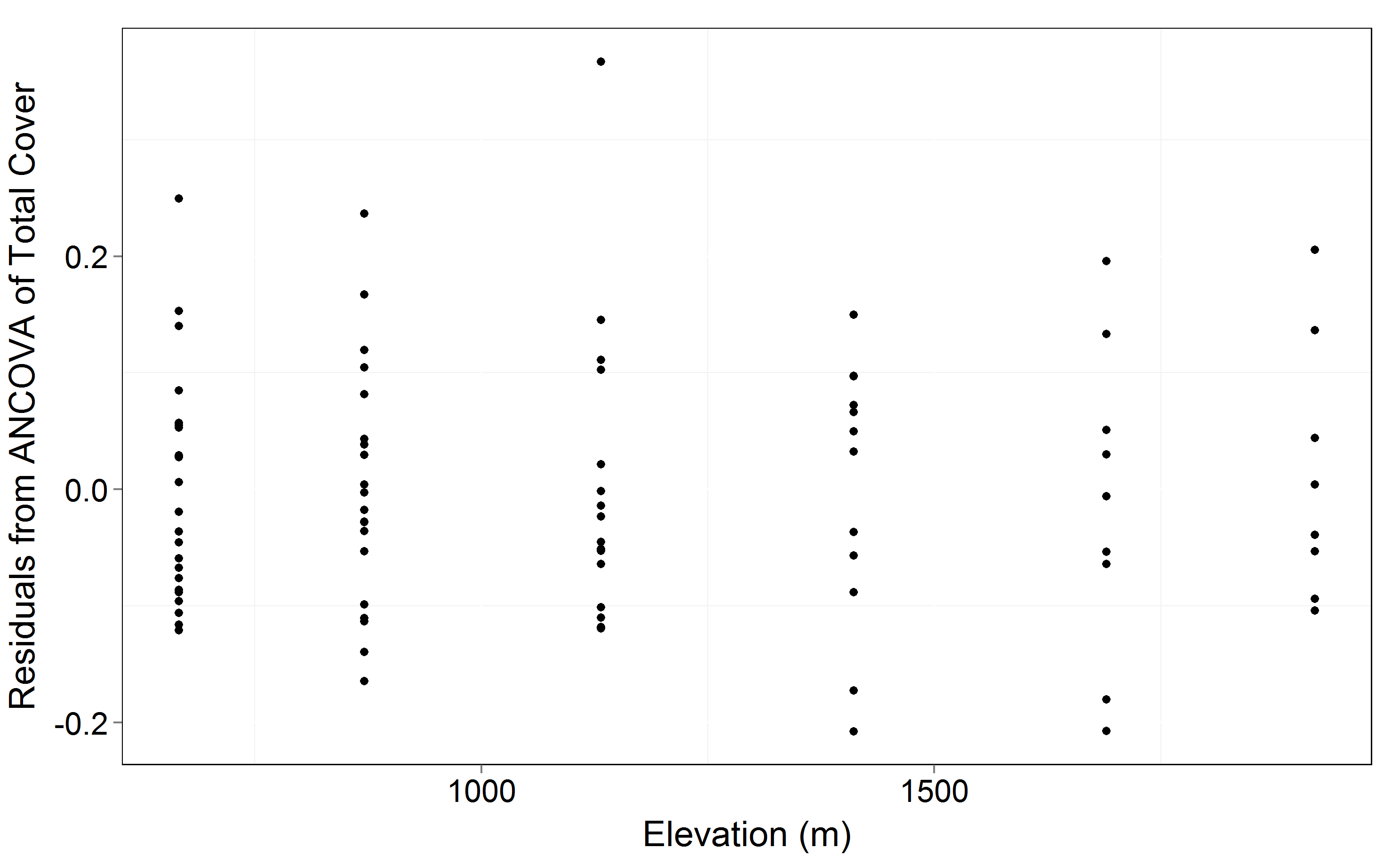

Supplement: Supplemental Information 3 — Figure shows the residuals from the ANCOVA analysis of total canopy cover against the different elevation sites were data were collected to determine if the ANCOVA model was a good fit for these data. [file peerj-02-477-s003.png]

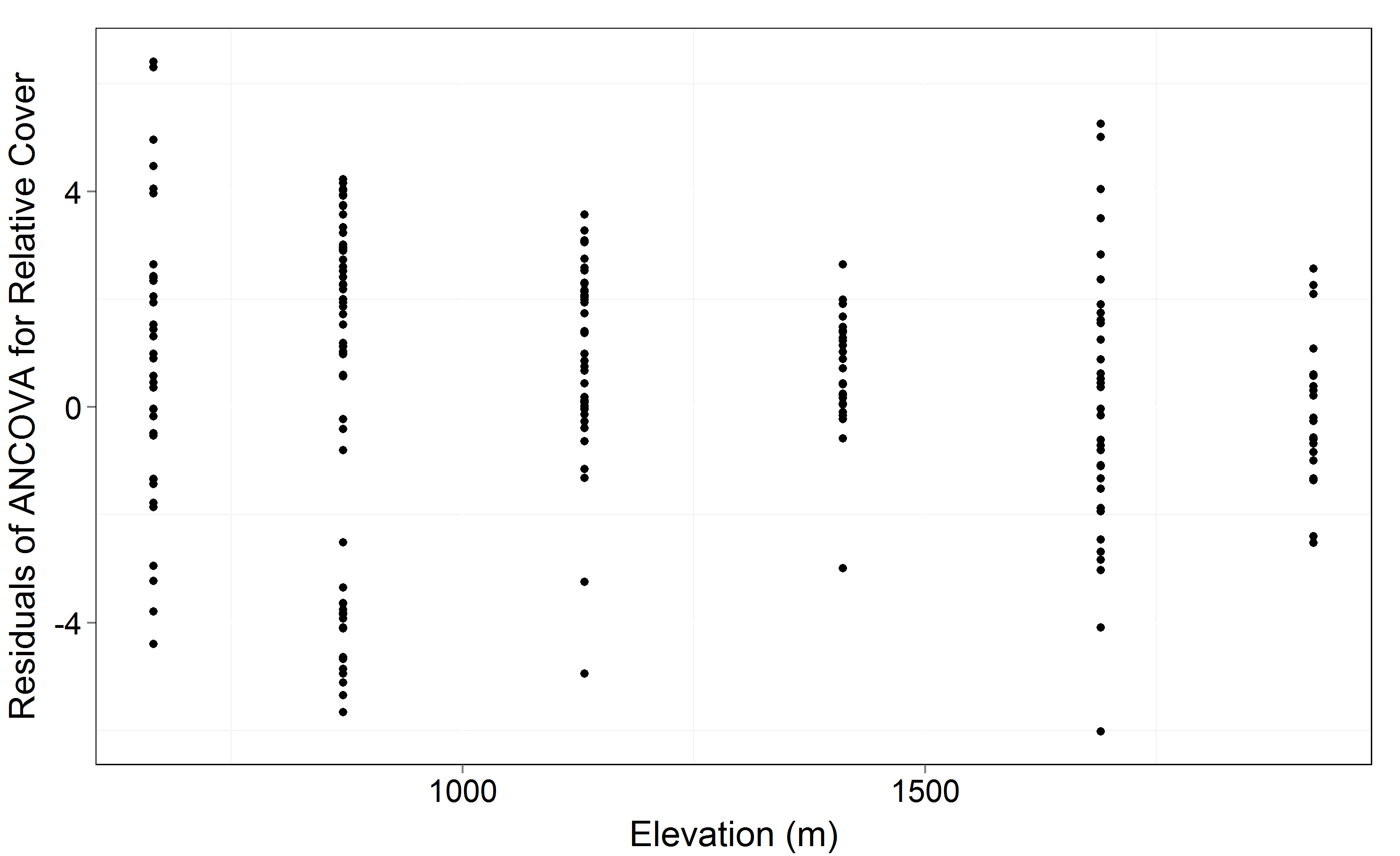

Supplement: Supplemental Information 4 — Figure shows the residuals from the ANCOVA analysis of relative cover against the different elevation sites were data were collected to determine if the ANCOVA model was a good fit for these data. [file peerj-02-477-s004.png]

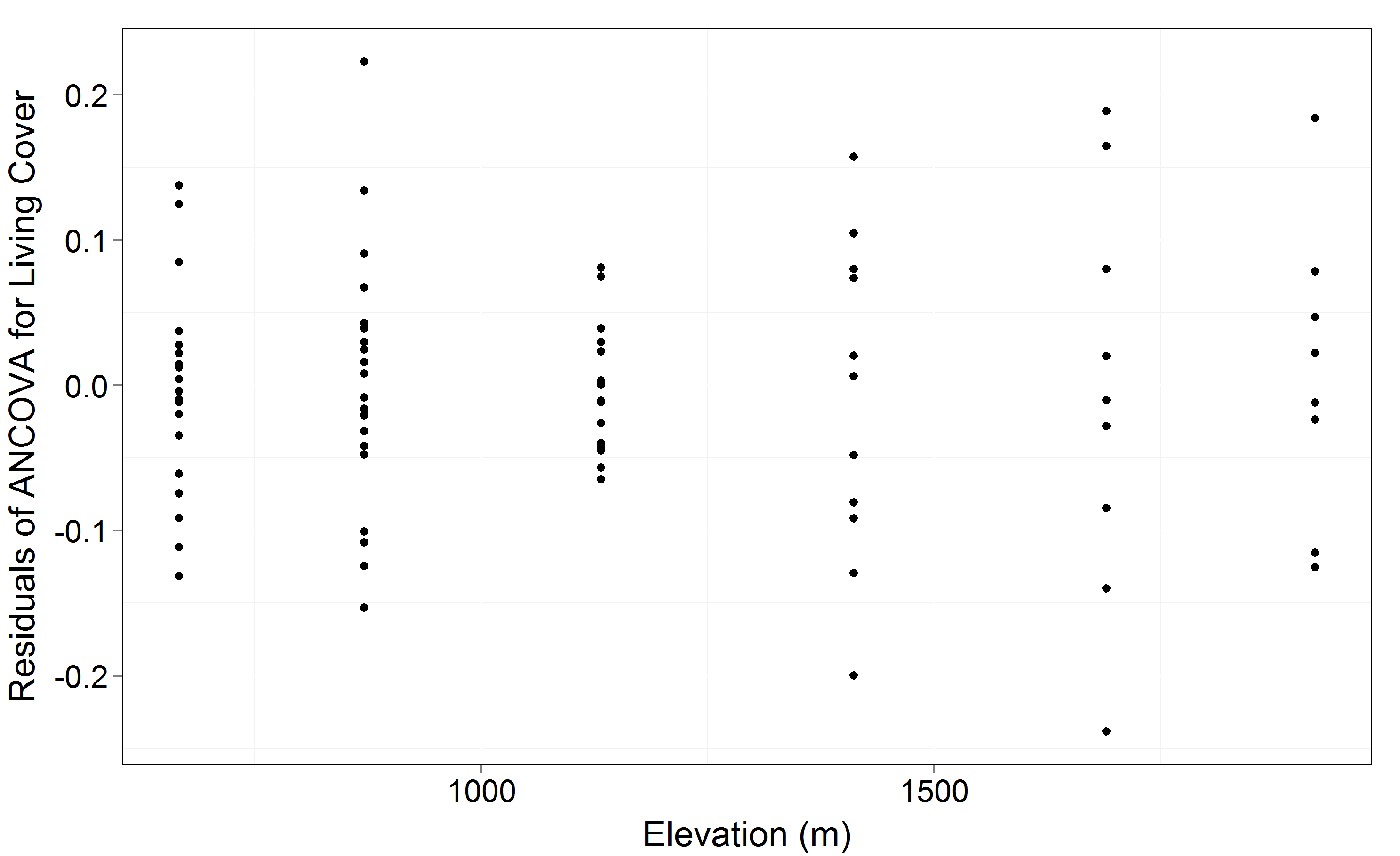

Supplement: Supplemental Information 5 — Figure shows the residuals from the ANCOVA analysis of living canopy cover against the different elevation sites were data were collected to determine if the ANCOVA model was a good fit for these data. [file peerj-02-477-s005.png]

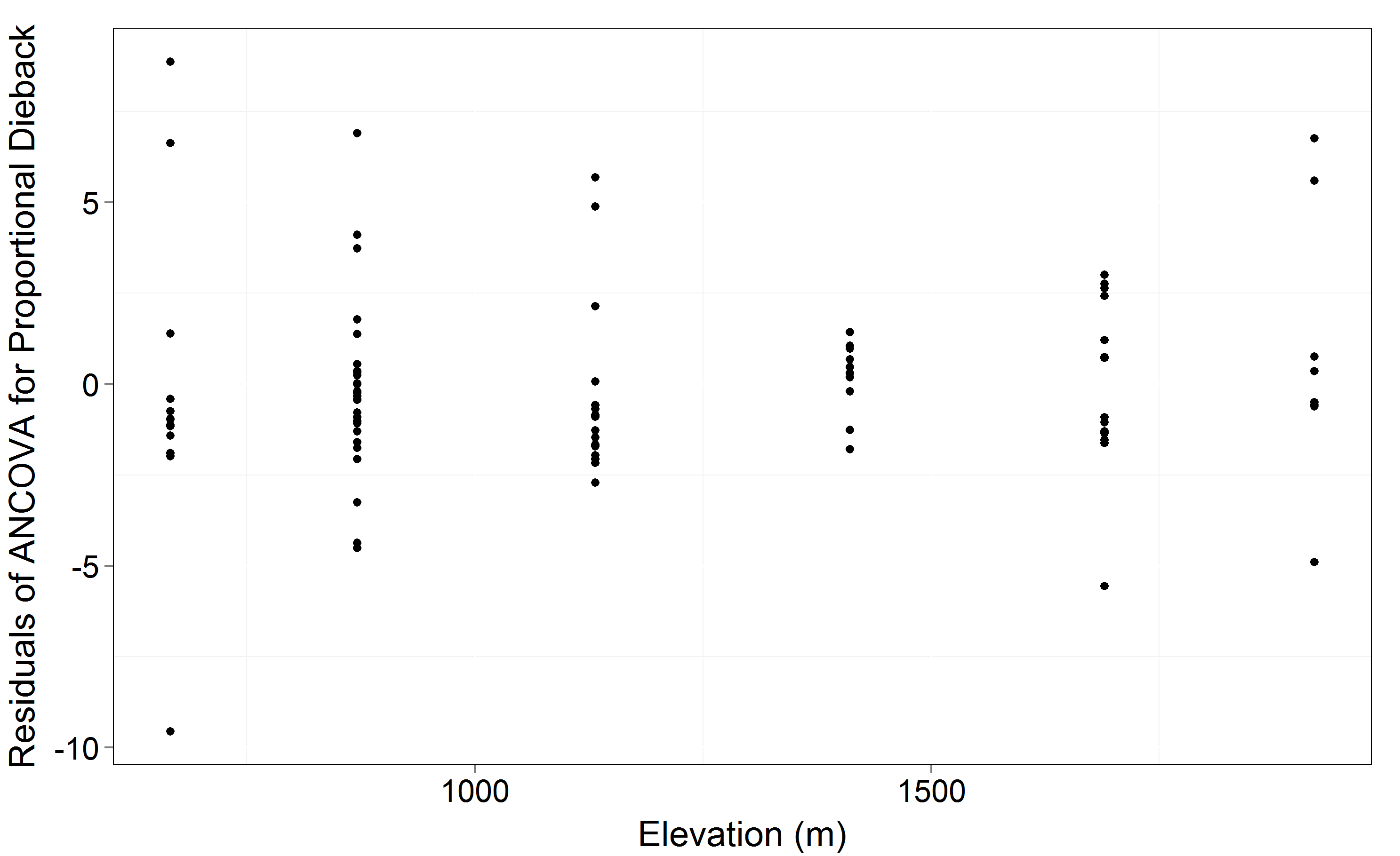

Supplement: Supplemental Information 6 — Figure shows the residuals from the ANCOVA analysis of logit transformed proportional dieback against the different elevation sites were data were collected to determine if the ANCOVA model was a good fit for these data. [file peerj-02-477-s006.png]
