# Supplementary material for: Plant dieback under exceptional drought driven by elevation, not by plant traits, in Big Bend National Park, Texas, USA
Source: PeerJ. 2014 Jul 15;2:e477. doi: 10.7717/peerj.477 (PMC4106195; doi:10.7717/peerj.477)
Supplement: Supplemental Information 2 — The mean proportional dieback for each species across elevations ±1 standard deviation. If there is no standard deviation shown with the proportional dieback for a particular species, there were <3 individuals measured at that location making it impossible to calculate a standard deviation. [file peerj-02-477-s002.docx]

| **Elevation** | **Species** | **Growth Form** | **Proportional Dieback** |
| --- | --- | --- | --- |
| 666 | *Fouquieria splendens* | shrub | 0.85±0.08 |
| 666 | *Krameria grayi* | shrub | 0.85±0.14 |
| 666 | *Larrea tridentata* | shrub | 0.85±0.13 |
| 666 | Unknown | shrub | 0.76±0.10 |
| 666 | Unknown | shrub | 0.81 |
| 666 | Unknown | subshrub | 0 |
| 666 | *Jatropha dioica* | subshrub | 0 |
| 666 | *Opuntia azurea var. parva* | subshrub | 0.35±0.47 |
| 666 | *Senna pilosior* | subshrub | 1.0±0 |
| 871 | *Acacia neovernicosa* | shrub | 0.29±0.26 |
| 871 | *Fouquieria splendens* | shrub | 0.20±0.23 |
| 871 | Unknown | shrub | 0.44±0.31 |
| 871 | Unknown | shrub | 0.37±0.13 |
| 871 | *Guaiacum angustifolium* | shrub | 0.52 |
| 871 | *Krameria grayi* | shrub | 0.42±0.13 |
| 871 | *Larrea tridentata* | shrub | 0.43±0.29 |
| 871 | *Leucophyllum candidum* | shrub | 0.31±0.16 |
| 871 | *Prosopis glandulosa* | shrub | 0.34±0.26 |
| 871 | *Jatropha dioica* | subshrub | 0.42±0.20 |
| 871 | *Opuntia azurea var. parva* | subshrub | 0.42±0.19 |
| 871 | Unknown | subshrub | 0.69 |
| 871 | *Agave lechuguilla* | succulent | 0.74±0.33 |
| 871 | *Dasylirion leiophyllum* | succulent | 1.0 |
| 871 | *Echinocereus dasyacanthus* | succulent | 1.0 |
| 871 | *Opuntia dulcis* | succulent | 0.82±0.06 |
| 871 | *Opuntia leptocaulis* | succulent | 0.60±0.44 |
| 871 | *Yucca torreyi* | succulent | 0.49±0.56 |
| 1132 | *Acacia greggii* | shrub | 0.82±0.08 |
| 1132 | *Dalea formosa* | shrub | 0.73 |
| 1132 | *Diospyros texana* | shrub | 0.68 |
| 1132 | *Ephedra torreyana* | shrub | 0.79±0.09 |
| 1132 | *Fendlera rupicola* | shrub | 0.75±0.08 |
| 1132 | *Guaiacum angustifolium* | shrub | 0.68 |
| 1132 | *Koeberlinia spinosa* | shrub | 0.79±0.16 |
| 1132 | *Leucophyllum candidum* | shrub | 0.79±0.05 |
| 1132 | Unknown | shrub | 0.84±0.06 |
| 1132 | *Mahonia trifoliolata* | shrub | 0.68 |
| 1132 | Unknown | shrub | 0.81 |
| 1132 | *Prosopis glandulosa* | shrub | 0.88 |
| 1132 | Unknown | shrub | 0.84 |
| 1132 | *Viguiera stenoloba* | shrub | 0.82±0.08 |
| 1132 | *Opuntia azurea var. parva* | subshrub | 1.0 |
| 1132 | Unknown | subshrub | 1.0 |
| 1132 | *Agave lechuguilla* | succulent | 0.90±0.11 |
| 1132 | *Dasylirion leiophyllum* | succulent | 0.86±0.11 |
| 1132 | *Opuntia dulcis* | succulent | 0.95±0.06 |
| 1132 | *Opuntia grahamii* | succulent | 1.0 |
| 1411 | *Acacia constricta* | shrub | 0.36±0.11 |
| 1411 | *Aloysia gratissima* | shrub | 0.35 |
| 1411 | *Dalea formosa* | shrub | 0.32±0.06 |
| 1411 | *Ephedra torreyana* | shrub | 0.36±0.11 |
| 1411 | *Fendlera rupicola* | shrub | 0.38 |
| 1411 | *Juniperus pinchotii* | shrub | 0.37±0.15 |
| 1411 | *Mahonia trifoliolata* | shrub | 0.39±0.09 |
| 1411 | *Mimosa aculeaticarpa* | shrub | 0.31±0.07 |
| 1411 | *Prosopis glandulosa* | shrub | 0.53 |
| 1411 | *Rhus microphylla* | shrub | 0.37±0.12 |
| 1411 | *Rhus trilobata* | shrub | 0.31±0.10 |
| 1411 | *Rhus virens* | shrub | 0.40±0.11 |
| 1411 | Unknown | shrub | 0.37±0.12 |
| 1411 | Unknown | shrub | 0.44±0.13 |
| 1411 | *Viguiera stenoloba* | shrub | 0.36±0.11 |
| 1411 | *Dasylirion leiophyllum* | succulent | 0.17±0.14 |
| 1411 | *Echinocereus dasyacanthus* | succulent | 0.02 |
| 1411 | *Nolina erumpens* | succulent | 0.15±0.15 |
| 1411 | *Opuntia chisosensis* | succulent | 0.17±0.14 |
| 1411 | *Yucca torreyi* | succulent | 0.25 |
| 1690 | *Acacia constricta* | shrub | 0.09 |
| 1690 | *Ericameria laricifolia* | shrub | 0.10±0.02 |
| 1690 | *Juniperus pinchotii* | shrub | 0.09±0.01 |
| 1690 | *Rhus trilobata* | shrub | 0.09±0.01 |
| 1690 | *Rhus virens* | shrub | 0.09±0.01 |
| 1690 | *Viguiera stenoloba* | shrub | 0.09±0.02 |
| 1690 | Unknown | subshrub | 0.29±0.20 |
| 1690 | *Agave havardiana* | succulent | 0 |
| 1690 | *Dasylirion leiophyllum* | succulent | 0 |
| 1690 | *Opuntia chisosensis* | succulent | 0 |
| 1690 | *Juniperus coahuilensis* | tree | 0.02 |
| 1690 | *Juniperus deppeana* | tree | 0.10±0.07 |
| 1690 | *Juniperus flaccida* | tree | 0.10±12 |
| 1690 | *Pinus cembroides* | tree | 0.09±0.08 |
| 1690 | *Quercus emoryii* | tree | 0.07±0.08 |
| 1690 | *Quercus grisea* | tree | 0.10±0.12 |
| 1920 | Unknown | shrub | 0.39±0.55 |
| 1920 | *Salvia regla* | shrub | 0.39±0.55 |
| 1920 | *Agave havardiana* | succulent | 0.04±0.08 |
| 1920 | *Dasylirion leiophyllum* | succulent | 0.04±0.08 |
| 1920 | *Opuntia chisosensis* | succulent | 0.04±0.08 |
| 1920 | *Juniperus deppeana* | tree | 0.05±0.03 |
| 1920 | *Juniperus flaccida* | tree | 0.06±0.03 |
| 1920 | *Juniperus coahuilensis* | tree | 0.03 |
| 1920 | *Pinus cembroides* | tree | 0.05±0.03 |
| 1920 | *Quercus emoryii* | tree | 0.05±0.03 |
| 1920 | *Quercus gravesii* | tree | 0.05±0.03 |
| 1920 | *Quercus grisea* | tree | 0.05±0.03 |
